# The recovery function of parasocial relationships for hopelessness on short-form video platforms: a moderated mediation study

**DOI:** 10.1186/s12889-025-24830-6

**Published:** 2025-10-30

**Authors:** Xinzhou Xie, Yanjun Lin, Qiyu Bai

**Affiliations:** 1 School of New Media, Peking University, Beijing, 100871 China; 2 State Key Laboratory of Media Convergence and Communication, Communication University of China, 100024, Beijing, China

**Keywords:** Hopelessness, Parasocial relationship, Perceived similarity, Self-identity, Well-being

## Abstract

**Background:**

Researchers have long examined the relationship between media use and negative psychological states. However, the correlation between hopelessness, parasocial relationships, and self-identity/ well-being on short-form video platforms is unknown. Additionally, the perceived similarity’s role in moderating these relationships has not been established.

**Methods:**

Drawing on the theoretical model for the development of parasocial relationships, we tested and confirmed the validity of a moderated mediation model, demonstrating how individuals experiencing hopelessness may obtain self-identity and well-being via the mediation of parasocial relationships and the moderation of perceived similarities on short-form video platforms.

**Results:**

The results of a survey among 2,902 users of short-form video platforms in China indicated that parasocial relationships mediated the relationship between hopelessness and self-identity/well-being. Perceived similarities had a significant moderation effect on the relationship between hopelessness and PSR and the indirect effect of perceived similarities on self-identity (or well-being).

**Conclusion:**

Users on short-form video platforms can autonomously derive positive health effects (self-identity/well-being) via PSRs. Perceived similarity exerted a moderating effect on the relationship between hopelessness and PSRs among individuals on short-form video platforms: the positive relationship between hopelessness and PSR is stronger for individuals with higher perceived similarities.

## Introduction

The concept of parasocial relationships (PSRs) has received considerable health communicaiton research interest. It was first proposed by Horton and Whol in 1956 [1] and can be considered as a long-term relationship between viewers and media personae that may initiate during viewing but endures beyond the media exposure context [2, 3]. Given that newly launched social media like short-form video platforms’ affordance of frequent self-disclosure and thus perceived intimacy and authenticity, the potential influence of media personae’s PSRs on the health of users of short-form video platforms is becoming increasingly profound.

No consensus has emerged, however, on the relationship between PSR and health in the context of short-form video platforms. Because short-form video platforms such as TikTok offer an effective connection between individuals, scholars have suggested that during the global pandemic, users could increase their intentions to exercise in parasocial experiences with fitness influencers on short-form video platforms [4]. According to Johnson et al. [5], however, individuals who establish PSRs with bloggers may be more inclined to buy alcohol following exposure to a pro-drinking blog post. Thus, owing to this duality and users’ need to avoid the negative connotations associated with the problematic use of short-form video platforms, it is necessary to explore whether and to what extent individuals in disadvantaged positions may report greater well-being in association with establishing PSRs on short-form video platforms, especially given that psychological issues have been aggravated in recent years by the global recession [6].

Hopelessness appears to be one type of negative psychological state; the concept of which refers to a negative psychological state in which individuals expect that negative things will occur in the future and feel they can do nothing about it [7]. Many individuals who experienced lockdown during the early stages of the COVID-19 pandemic suffered from hopelessness because the virus was unknown, and their futures had become unpredictable [8]. Since hopelessness is the strongest predictor of depression severity and suicidal behavior, it is imperative to intervene in hopelessness through external means [7]. However, the association between hopelessness and media use has not yet been fully studied. Thus, this study aimed to explore how individuals experiencing hopelessness may be able to cope with its negative impact through PSRs formed via short-form video platforms. To further distinguish itself from these and other studies about social media and health, this study focuses on psychological health—specifically self-identity and well-being—in relation to PSRs, rather than on health behaviours such as healthy eating [9] or intention to exercise [10], which inherently explore the association between PSRs and behaviours.

The present study also underscored the links between users’ autonomy and PSRs by focusing on the moderating variable of perceived similarity (i.e., the extent of similarity between the viewer and the viewed, as perceived by the viewer). Numerous empirical studies have reported that perceived similarity is an important positive antecedent to PSRs in prior social media platforms [11, 12]. For vulnerable individuals in particular, higher levels of homophily can reduce uncertainty and foster the development of PSRs with media personae, ultimately contributing to a sense of belonging and achievement [13, 14]. As media personae become increasingly present and available, it is essential to reconceptualize the moderating role of perceived similarity during the PSR process when individuals use short-form video platforms and to offer more nuanced insights into this phenomenon.

This study advances the theoretical understanding of PSRs by exploring how individuals experiencing hopelessness, as a user type, can improve subjective agency to achieve their self-identity/well-being through PSRs on social media. Furthermore, it offers valuable insights into how the unique interaction mechanisms of short-form video platforms can empower PSRs, especially how perceived authenticity in short-form videos can increase users’ sense of perceived similarity and ultimately shape the way hopeless audiences engage with PSRs.

## Theoretical background and hypotheses development

### The interactive nature of short-form video platforms

Launched in 2016, short-form video platforms such as Douyin (the Chinese version of TikTok) have enjoyed seemingly unstoppable success. As of June 2022, they had over 962 million users, encompassing 91.5% of Chinese netizens [15]. Two examples of giants in the Chinese short-form video market are Douyin and Kuaishou. Douyin focuses on audiences in first- and second-tier cities such as Beijing and Shanghai, while the target audiences of Kuaishou are citizens of second and third-tier cities and those in rural areas [16]. These mobile-based apps mainly provide video content that is shorter than 5 min.

Although prior social media can also provide short-form videos, current short-form video platforms focus on users’ participation and the replicability of the content production. Three important distinguishing characteristics of short-form video platforms should be noted. First, in terms of design, the “shooting” button is located at the bottom center of the interface, which allows users to start recording a video at any time. This function encourages users to participate in the production process.

Second, compared with other social network sites, short-form video platforms’ communication models center on memes. Specifically, they rely on imitation as social currency rather than chats by offering plentiful, easy-to-use pre-formed templates [17]. These templates typically provide sampled material such as audio and emoji memes; Hashtags can also constitute memes [18]. Users need only pick their preferred template and upload their photos or videos into the template. The apps process the photos or videos and automatically generate and edit a final video. For example, many Chinese users used Bing Dwen Dwen mascot memes during the run-up to the 2022 Winter Olympics: they made videos in which they were wearing the Bing Dwen Dwen headdress special effect by placing their face in Bing Dwen Dwen memes. A pre-formed template like this may well have made disparate users feel connected.

Livestreaming can also function as an important communication tool. In this media form, users can acquire more knowledge about media personae through self-disclosure (e.g., talking about their personal experiences at work or in their family life) [19]. Additionally, viewers can easily approach media personae nearly synchronously during live events when there are not too many other viewers, thus facilitating instant responses from given media personae. Mutual awareness can therefore be invoked, and users’ psychological needs can be met via such a seemingly equal establishment of online interaction with otherwise unreachable media personae [20]. Researchers have found, for example, that TikTok users who were mourning felt connected and comforted when they engaged with a live host [21].

Given these distinguished functionalities, interactions on short-form video platforms can be seen as a creative form of online interaction: users are encouraged to follow media personae for a long time and build relationships with them through various, seemingly two-way, communication modes (e.g., participating memes and livestreaming).

### Hopelessness and media use

Hopelessness constitutes two main elements: (a) pessimistic future-oriented thinking and (b) the perception of an impassable impediment to goal achievement [22]. Much media-related research has correlated this negative mental status with virtual interactions in a given medium. For example, scholars have long been concerned with the possibility that improper media use can cause negative outcomes such as hopelessness and depression [23]. Kubey [24] suggested that aging viewers who are exposed to negative depictions of older persons may begin to feel unwanted.

However, there is also evidence suggesting that the association between media use and psychological issues could be reversed. Especially since the 1970 s, numerous uses and gratifications studies have suggested that users’ characteristics and purposes for consuming media play a crucial role in media involvement [25, 26]. Consequently, vulnerable individuals may seek virtual relationships (PSRs) as functional alternatives. For example, previous empirical evidence suggests that individuals experiencing loneliness can approach PSRs for help and thereby fulfill their interpersonal needs [27]. This is because the virtual space provided by media can help individuals who are vulnerable and sensitive to rejection to enjoy the experience of real social interactions [28]. In this respect, vulnerable audiences may turn to viewer-performer relationships as a means of engaging with issues related to identity and self-concept, sometimes encountering experiences they have not had in real life [29].

Nevertheless, an understanding of the ways in which hopeless individuals’ motivations can generate positive aspects of self-concept formation—such as well-being and self-identity via the viewer-performer bond when individuals use short-form video platforms—is still missing. The present study addressed this gap in the literature by exploring the underlying processes of these connections.

### Hopelessness and PSRs on short-form video platforms

As previously mentioned, examinations of the association between deficit viewers and media personae indicate that the parasocial experience is the most observed phenomenon in virtual interactions [27]. Horton et al. [1] first used the term “parasocial interaction” to describe a one-sided relationship between users and media personae that exists in the user’s mind as a belief that they have direct interactions with one other. Later, parasocial researchers expanded the concept and differentiated between parasocial interactions (PSIs) and parasocial relationships (PSRs). Specifically, whereas PSIs can be defined as imagined interactions that only occur during media exposure, PSRs refer to deeper relationships that may start during viewing but persist beyond the context of media exposure [2, 30]. That is, in contrast to a PSI, a PSR tends to be more similar to real relationships with enormous investments in time, emotion, or money [31]. Once users have established a PSR, it does not disappear even if the users temporarily stop paying attention to the media personae; it remains in the users’ minds and is ready to provide help to users whenever they need it. It has thus been suggested that health-related research should focus more on PSRs than PSIs because users can obtain more potentially positive effects from engagement with media personae in such enduring relationships [32].

Because parasocial relationships (PSRs) resemble face-to-face social interactions, research on PSRs often draws from interpersonal communication theory, particularly the substitution hypothesis [33]. A notable advancement in this area is the theoretical model of the development of parasocial relationships proposed by Tukachinsky et al. [34], which synthesizes sixty years of PSR studies and incorporates Knapp’s model of relationship development [35, 36]. Unlike earlier studies that often treated PSRs as static constructs or one-time reactions, this model conceptualizes PSRs as dynamic, evolving experiences. It is also the first to place PSRs at the center of analysis, offering a comprehensive framework that captures the full trajectory of PSR development [31, 37].

According to this theory, the motivation of satisfying special needs serves as an antecedent to the formation of PSRs [34]. Specifically, users who experience feelings of helplessness or low self-worth in real life may actively seek out media content that provides comfort, encouragement, or validation. For example, someone experiencing helplessness may be compelled to turn to PSRs for help [38]. Importantly, this PSR is particularly likely to occur between users and the media personae they previously followed. This is because these media personae are more trusted for inferior individuals. Thus, PSRs with media personae, especially those whom users have been constantly following, can be used as meaningful tools for individuals with low self-evaluation and negative moods: they can help those who feel hopeless overcome mental and emotional challenges [39].

This process of engaging with PSRs as a way to navigate or mitigate feelings of hopelessness may be especially facilitated on short-form video platforms, given their distinctive patterns of interaction. The theoretical model of the development of PSRs asserts that accessibility (the extent to which media personae would like to interact with users) has great potential to shape users’ experience of closeness in such relationships [34]. For example, live-streaming allows short-form video users to engage in more meaningful communication with media personae; during live events, viewers can directly communicate with media personae and participate in quasi-synchronous conversations [40]. As such, hopeless individuals may regard short-form video platforms as a means of attaining comfort: they may seek help from vloggers with whom they develop PSRs owing to the similarities between PSRs on short-form video platforms and real-life social relationships. Therefore, we proposed the following hypothesis:

- Hypothesis 1 The more individuals feel hopelessness, the stronger the PSRs they will build.

### PSRs and self-identity/well-being

Self-identity is conceptualized as a core component of individual cognitive processes in cognitive psychology [41]. This concept focuses on how a developing individual endowed an attitude toward himself -or herself [42]. For example, whether or not “the individual feels certain about what he/she should do with her life” and “feels proud to be the sort of person he/she is”. If the positive outweigh the negative, individuals are better equipped to handle future difficulties [43]. As such, self-identity as cognition becomes a crucial element of mental health because it can guide individuals’ behaviors, especially when encountering difficulties [44].

Self-identity is traditionally formed through the social groups [45]. In the social media era, however, individuals’ self-identity is increasingly shaped not only by interpersonal interactions but also by mediated experiences—especially sustained engagement with media personae. A key pathway for the role of mediated experiences on self-identity lies in the activation and intensification of PSRs, through which users interact with media personae in socially meaningful ways that shape self-perception and identity [46].

According to the theoretical model of the development of parasocial relationships, as a PSR intensifies, users develop strong trust in the media persona and reduce psychological resistance to their messaging [34, 47]; Over time, these figures may be internalized as role models, shaping users’ opinions and self-understanding [48]. This cognitive alignment suggests that stronger PSRs can be associated with users’ self-identity by offering emotional resonance and behavioral guidance.

Building on this foundation, recent research suggests that short-form video platforms like TikTok may further amplify these effects. With affordances such as algorithmic curation, short-form storytelling, and high-frequency self-disclosure, these platforms foster repeated exposure to familiar media personae. Such features have facilitated a shift from the “networked self” to the “algorithmic self,” where identity is no longer solely formed through social dialogue but through recursive interactions with algorithm-selected representations of the self [49]. As algorithms continuously surface media personae aligned with users’ prior behavior, they reinforce perceived intimacy and similarity—key elements in PSR reinforcement. In this context, PSRs become both emotionally and algorithmically conditioned, deepening their influence on users’ evolving sense of self. These effects may be further enhanced by immersive features such as livestreaming and participatory culture (e.g., memes, co-creation), which promote sustained engagement and co-construction of meaning between users and media personae. In light of these dynamics, we proposed the following hypothesis:

- H2a: The stronger the PSRs between individuals and media personae, the more positive self-identity the individual feels.

As an important branch of media psychology research, parasocial research also explores how parasocial experiences enhance user well-being through various media [50]. In general, well-being is conceptualized through two major paradigms: hedonic and eudaimonic. The hedonic approach focuses on subjective well-being, emphasizing individuals’ evaluations of their lives based on positive affect, low negative affect, and a sense of life satisfaction [51]. In contrast, the eudaimonic paradigm emphasizes functional well-being, referring to individuals’ capacity to “do well” in life—through personal growth, purpose, autonomy, and self-actualization—rather than simply “feel good” [52]. Despite this distinction, media scholars often adopt an integrative perspective that combines both paradigms. For instance, Keye describes well-being as “flourishing” [53], while Seligman [54] refers to it as living “a full life.” These integrated notions correspond closely with emotional outcomes associated with PSRs: on one hand, increased happiness and satisfaction may arise from affective investment in media personae [27]; on the other hand, deeper feelings of purpose, meaning, and belonging may emerge from long-term parasocial bonds [55].

As previously noted, short-form video platforms offer novel forms of relationship; therefore, it is worthwhile to investigate how PSRs can fulfill users’ need to improve their aspirational capability. For example, by posting memes rather than reading comments, viewers may find it easier to foster a sense of belonging and enhanced emotional effects [21]. Therefore, we proposed the following hypothesis:

- H2b: The stronger the PSRs between individuals and media personae, the more well-being the individual feels.

The term PSR implies an online, one-sided, long-term relationship between media personae and viewers [3]. According to PSR theory, such relationships typically evolve through two main stages [56]. In the first stage, viewers’ psychological needs interact with media personae’s self-disclosure, fostering perceived intimacy through curated and authentic content [31]. In the second stage, PSRs may reduce viewers’ reactance and counterarguing, thereby influencing their cognition and emotion [34]. That is, PSRs act as an important mediator between motivations and attitudes or psychological feelings. While several empirical studies have identified these two processes [10, 57, 58], none has focused on the psychological effects of PSRs among users of short-form video platforms experiencing hopelessness. Having acknowledged the unique features of short-form video platforms in terms of self-disclosure, patterns of interactions, and memes [40], users experiencing experiencing hopelessness may turn for help to PSRs with vloggers whom they closely follow for help. Such a PSR can also be related to positive outcomes for the user’s self-identity/well-being. Therefore, we proposed the following hypothesis:

- H3a-b: PSR mediates the relationship between hopelessness and – in particular, (a) self-identity and (b) well-being.

### Perceived similarity and PSRs

Perceived similarity refers to an individual’s subjective perception that another individual is similar to them [59]. The theoretical model of the development of parasocial relationships indicates that viewers’ perceptions of similarity between them and given media personae can affect their PSR process [34]. Media users may record the quality of relationship schemata with media personae they encounter, creating a mental representation of their relationship that includes similarities between them (the viewer) and media personae [60]. These relationship schemata will be activated and recalled before the formation of PSRs. If the relationship schemata fit viewers’ situational needs, the likelihood of continuing the relationships with the corresponding media personae will increase [61].

This explanation is related to the similarity-attraction mechanism. Specifically, individuals—especially those who are vulnerable—tend to choose to make connections with strangers who seem similar to them on first acquaintance [13]. This is because the perception of similarities reduces the time cost required for accepting new ideas, and therefore increases the effectiveness of subsequent communication between media personae and viewers. Prior studies have indicated that this pattern also applies to social media [12]. For example, participants who held similar political beliefs to Trump were found to be more susceptible to forming parasocial relationships with him and show greater agreement with his viewpoints on social media [62]. Because individuals experiencing hopelessness are more vulnerable and have a stronger yearning for comfortable and meaningful communication, the positive association between hopelessness and PSRs may be amplified among those who perceive greater similarity. Therefore, we proposed the following hypotheses:

- H4: Perceived similarity positively moderates the relationship between hopelessness and PSRs, so that the positive relationship between hopelessness and PSR is stronger for individuals with higher perceived similarities.
- H5a-b: Perceived similarity moderates the indirect effect of hopelessness on (a) self-identity and (b) well-being via PSRs, such that the more similarities individuals perceive, the stronger the positive relationship between hopelessness and self-identity/well-being.

**Fig. 1:**
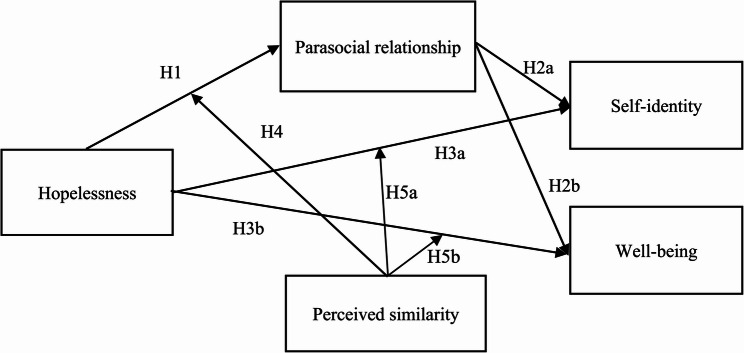
Proposed research model with all hypotheses

The proposed research model with all the hypotheses can be seen in Fig. 1.

## Methods

### Procedures

We collected survey data in March 2021 via the survey research firm, Ipsos. We used quota sampling stratified by the age, gender, and educational level of Chinese netizens. According to the 47th Statistical Report on China’s Internet Development, 88.3% of China’s total netizens are short-form video platform users, and the user composition is essentially the same as that of the overall Internet users [63]. To to enhance the representativeness of the sample, we improved the accuracy of quotas by using user information from data reports of the two main Chinese short-form video platforms (Douyin and Kuaishou). Volunteers were asked to complete a computerized self-report questionnaire. The demographic structure of the final sample corresponds to the basic composition of Chinese short-form video platform users.

In the questionnaire, we gave a precise description of each variable. For example, the media personae with whom users had built PSRs referred to vloggers they had been following regularly on short-form video platforms and whom the users viewas real friends. We asked respondents to name vloggers whom they consistently follow on short-form video platforms and informed participants that the subsequent questions were about their relationship with their closely followed vloggers. We also stressed that short-form videos offered by media personae were inspirational and informative rather than entertaining or a form of escapism.

To ensure the quality of the outcome, questionnaires from 98 respondents were eliminated on the basis of their responses to attention-checking questions (e.g., “The earth is square”). The final sample size was 2,902 (48% female) with a mean age of 31.5 years (SD = 9.71). Most short-form video users regularly use at least one of the two major platforms available in China. All participants were guaranteed anonymity, and their informed consent was obtained before commencing the online survey.

### Measures

#### Hopelessness

We used six items based on Li et al.’s [64] Chinese version of the hopelessness scale, which was adapted from a study by Bolland et al. [65]. On a 5-point Likert-type scale, respondents were asked to rate their agreement with each of the six items; for example, “I don’t have good luck now, and there is no reason to expect that I will when I get older” (Cronbach’s α = 0.923).

#### Parasocial relationship

The measures used to evaluate the interaction process between viewers and media personae were adapted from Rubin et al. [27]. The 5-point Likert-type scale included 14 items instead of 20, as only those capturing the core traits of user–media personae relationships on short-form video platforms were retained.; for example, “I like to compare my ideas with what media personae said in the video clip” (Cronbach’s α = 0.897).

#### Self-identity

The brief self-identity scale by Ochse et al. [43] was used to assess users’ self-identity. Five items of this scale concerning evaluations of self rather than perceptions of external judgments were selected, translated and adapted into Chinese For example, “I feel certain about what I should do with my life,” The items were rated on a 4-point scale from “never” to “very often” (Cronbach’s α = 0.718).

#### Well-being

To measure well-being, we used the 20-item Chinese scale developed by Xing [66]. The scale comprises 10 dimensions, including target value and self-acceptance. Items included, for example, “Most of my life goals inspire me rather than discourage me.” The items were assessed on a 6-point Likert-type scale ranging from “strongly disagree” to “strongly agree” (Cronbach’s α = 0.819).

#### Perceived similarities

 The moderator variable that we chose to examine was perceived similarities, which was assessed by averaging 3 adapted items from Auter et al. [67], such as, “I seem to have the same beliefs and attitudes as the character. Items like “I’d enjoy interacting with media personae and my friends at the same time” was removed from the original version of the scale due to conceptual is unrelated to intrinsic values.(Cronbach’s α =.689).

Given that age, gender, and educational level were commonly selected as control variables in both hopelessness literature [68, 69] and parasocial relationship research [70, 71], we controlled these demographic variables in the present study.

### Statistical analysis

First, descriptive statistics and a correlation matrix were generated. Second, the PROCESS macro (Model 4), developed by Hayes [72], was used to test the mediation effect of a PSR between hopelessness and self-identity/well-being. Third, the PROCESS macro (Model 7) was used to examine whether perceived similarities moderated these mediation processes. We also employed the bootstrapping method, which processed 95% bias-corrected confidence intervals (CIs) from 5,000 data samples to ensure the significance of indirect effects [73]. The results indicated that these effects were significant when the CIs excluded zero.

#### Preliminary analysis

Descriptive statistics (means, standard deviations, and zero-order correlations) were generated for all the variables and are presented in Table 1. The internal consistency alphas are also shown in the table. The results showed that the correlation between hopelessness and well-being and between hopelessness and self-identity were both negative.

**Table 1:** Descriptive statistics, alpha coefficients, and correlations

	M	SD	1	2	3	4	5	6	7		8
1.Gender	-	-									
2.Age	31.50	9.714	− 0.129**								
3.Education	3.060	1.044	0.171**	− 0.478**							
4.Hopelessness	2.719	1.095	− 0.189**	0.161**	− 0.327**	(0.923)					
5.PSR	3.611	0.614	− 0.062**	0.048*	− 0.086**	0.218**	(0.897)				
6.Well-being	4.473	0.742	0.106**	− 0.034	0.214**	− 0.653**	0.105**	(0.819)			
7.Self-Identity	3.021	0.494	− 0.066**	0.049**	0.020	− 0.045*	0.546**	0.389**		(0.718)	
8. PS	3.618	0.753	0.018	− 0.028	− 0.030	0.183**	. 0.685**	037*		0.382**	(0.689)

*N* = 2,902. Internal reliabilities (alpha coefficients) for the constructs are given in parentheses on the diagonal

*PSR *parasocial relationship,* PS* perceived similarity

*p <.05, **p <.01

#### Simple mediation analysis

According to Model 1 (Table 2), hopelessness was positively related to PSR (B = 0.210, SE = 0.020, *p* <.001). Hence, Hypothesis 1 was supported. According to Model 2 (Table 2), PSR was positively associated with self-identity (B = 0.583, SE = 0.016, *p* <.001). Therefore, Hypothesis 2a was supported.

**Table 2:** Testing the mediation effect of PSRs on the relationship between hopelessness and self-identity/well-being

Predictors	Model 1 (PSR)		Model 2 (self-identity)		Model 3 (well-being)	
	B (SE)	t	B (SE)	t	B (SE)	t
Age	0.001 (0.002)	0.286	0.007 (0.002)	3.919***	0.010 (0.002)	6.043***
Gender	− 0.040 (0.037)	−1.077	− 0.127 (0.031)	−4.086***	− 0.018 (0.027)	− 0.672
Education	− 0.011 (0.021)	− 0.526	0.053 (0.017)	3.085**	0.048 (0.015)	3.158**
Hopelessness	0.210 (0.020)	10.824***	− 0.177 (0.017)	−10.680***	− 0.710 (0.014)	−49.415***
PSR			0.583 (0.016)	37.502***	0.259 (0.014)	19.203***
*R* ^*2*^	0.048		0.335		0.498	
*F*	36.743***		291.563***		573.353***	

*N* = 2, 902. Each column is a regression model that predicts the variable at the top of the column

*PSR *parasocial relationship

*p <.05, **p <.01, ***p <.001

The results also showed that hopelessness had an indirect effect on self-identity (B = 0.122). Bootstrapping results confirmed the significance of the indirect effect, with a 95% CI [0.097, 0.148]. Therefore, Hypothesis 3a was also supported.

Regarding the path among hopelessness, PSR, and well-being, the results shown in Model 3 (Table 2) illustrate that PSR was positively associated with well-being (B = 0.259, SE = 0.014, *p* <.001; see Model 3 in Table 2). Therefore, Hypothesis 2b was supported. Furthermore, the results indicated that hopelessness had an indirect effect on well-being (B = 0.054). Bootstrapping results confirmed the significance of the indirect effect, with a 95% CI [0.042, 0.068]. Therefore, Hypothesis 3b was also supported.

#### Testing for a moderation effect

The results relating to Hypothesis 4 are reported in Table 3. The results demonstrated that the interaction between hopelessness and perceived similarity moderated PSR (B = 0.040, *p* <.001; see Table 3), which supported Hypothesis 4.

**Table 3:** Testing the moderated mediation effect of perceived similarity on self-identity/well-being

Predictors	Model 4 (PSR)		Model 5 (self-identity)		Model 6 (well-being)	
	B (SE)	t	B(SE)	t	B (SE)	t
Age	0.004 (0.002)	2.725**	0.007 (0.002)	3.919***	0.009 (0.002)	6.043**
Gender	− 0.101 (0.028)	−3.682***	− 0.127 (0.031)	−4.0856***	− 0.019 (0.027)	− 0.672
Education	− 0.012 (0.015)	− 0.816	0.053 (0.017)	3.085**	0.048 (0.015)	3.158**
Hopelessness	0.064 (0.015)	4.272***	− 0.177 (0.017)	−10.680***	− 0.710 (0.014)	−49.415***
PS	0.677 (0.014)	49.43***				
Hopelessness × PS	0.040 (0.013)	3.125**				
PSR			0.582 (0.016)	37.502***	0.260 (0.013)	19.203***
*R* ^*2*^	0.485		0.335		0.498	
*F*	454.258***		291.563***		573.353***	

*N* = 2,902. Each column is a regression model that predicts the variable at the top of the column

*PS *perceived similarity

*p <.05, **p <.01, ***p <.001

**Fig. 2:**
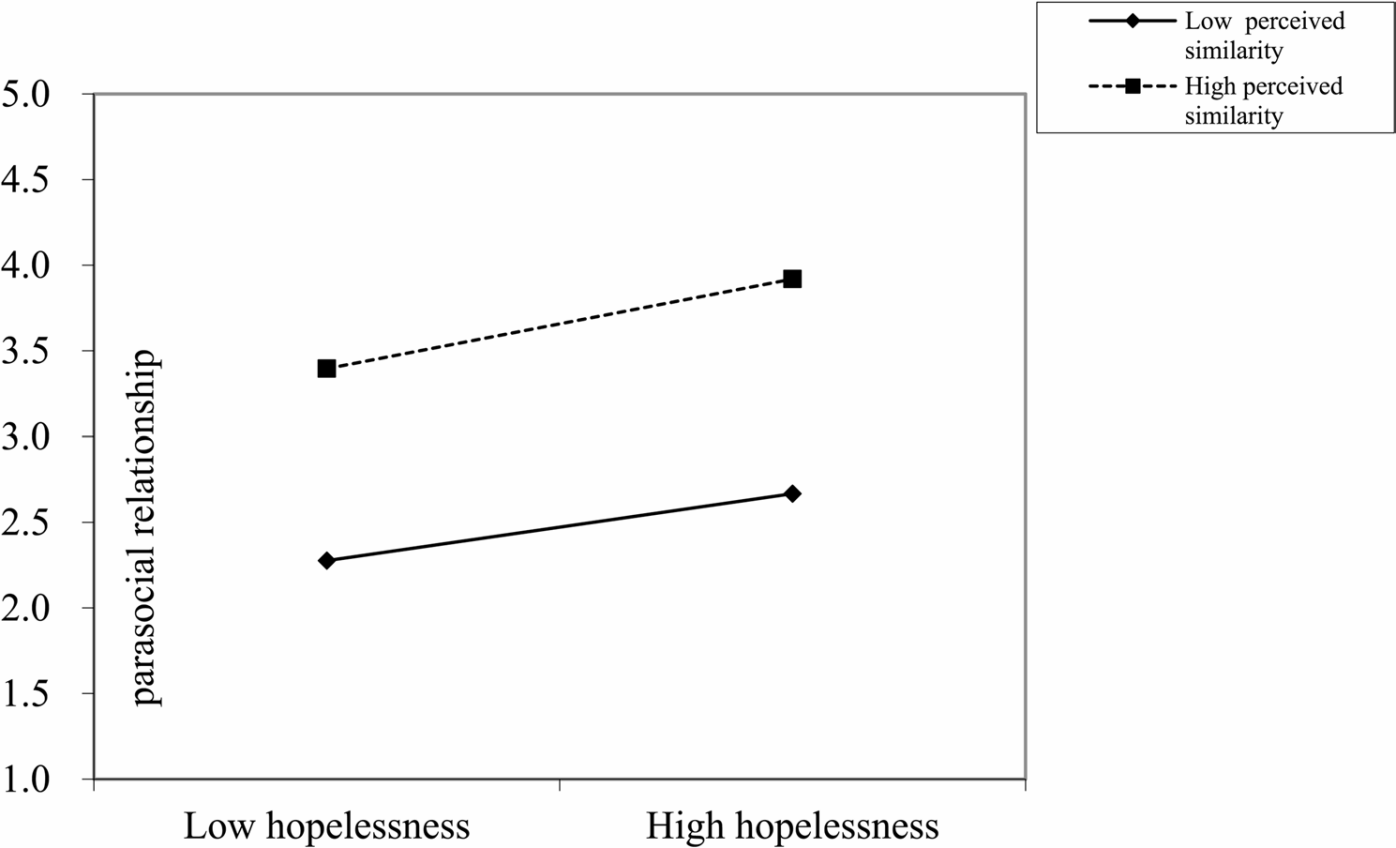
The interaction effect of hopelessness and perceived similarities on PSRs. High and low levels of hopelessness and perceived similarities represent 1 standard deviation above and below the mean, respectively

We then plotted a slope to illustrate the relationship between hopelessness and PSR, for low and high levels of perceived similarity, respectively. As Fig. 2 shows, the slope of the relationship between hopelessness and PSR with high perceived similarity was significant (B _high perceived similarity_ = 0.239, t = 3.770, *p* <.001). Meanwhile, the slope was comparatively weaker among those individuals who perceived low similarity (B _low perceived similarity_ = 0.179, t = 4.164, *p* <.001). Thus, Hypothesis 4 was supported.

**Fig. 3:**
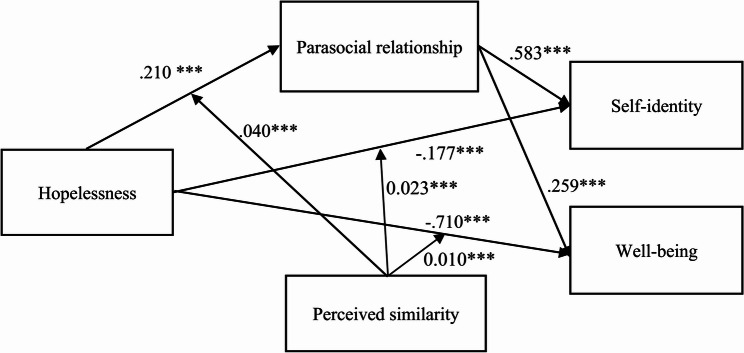
Summary results of the moderated mediation analysis

The results indicated that the index of moderated mediation on self-identity was significant (B = 0.023, SE = 0.009, 95% CI = [0.004, 0.043]), and the index of moderated mediation on well-being was also significant (B = 0.010, SE = 0.004, 95% CI = [0.002, 0.020]). That is, the indirect effect of hopelessness on self-identity (or well-being) was stronger for individuals with a high level of perceived similarities than those with a low level of perceived similarities. Thus, hypothesis 5 was supported. Summary results of the moderated mediation analysis are illustrated in Fig. 3.

## Discussion

This study aimed to examine the psychological health among hopeless individuals on short-form video platforms, with a specific focus on exploring the relationship among hopelessness, PSRs, and self-identity/well-being. Our findings provide insight into the ways in which hopeless user’s agency affects communication practices and enrich the theoretical model of the development of parasocial relationships in the health field in the short-form video platform context.

The foregoing results illustrate that a person’s hopelessness positively relates to their self-identity/well-being through PSRs formed via short-form video platforms. This result is integrated into the logic of PSR theory and emphasizes the positive effects generated by virtual relationships conducted via this new social media format. Furthermore, under the moderation effect of perceived similarities, the positive relationship between hopelessness and PSR is stronger for individuals with higher perceived similarities.

### Theoretical implications

The present study makes several theoretical contributions to the literature. First, we observed that hopelessness positively affects the strength of PSRs on short-form video platforms, which expands research on the association between social media and mental health. More specifically, while previous research has focused on psychological outcomes such as stress and anxiety, this study introduces hopelessness, a predictor of psychological outcomes, as a new variable. Compared with other severe psychological constructs derived from latent harmful childhood experiences, hopelessness often emerges from an individual’s self-attributions and negative self-conceptions [7]. These perceptions can largely be overcome with appropriate social support [74]. In other words, it is likely that individuals experiencing hopelessness are capable of improving their situation with external help and can benefit from effective interventions to prevent depression. As individuals experiencing hopelessness use short-form video platforms, they can actively acquire social support from media personae, often by increasing the frequency of their interactions with the media personae they follow constantly through various forms of interaction and different types of content. Therefore, the present study emphasizes the potential role of hopelessness as a new variable in media interaction research, which can play a positive role in preventing mental health disorders.

Second, this study enriches the research on the relationship between PSRs and mental health by developing novel interaction paradigms. PSRs positively relate to self-identity and well-being on short-form video platforms: the stronger the PSR, the stronger the user’s sense of identity and perceived well-being. The explanation is related to the expanded scope of media personae. PSRs with mediated nonfictional characters were originally referred to as “macro celebrities” (e.g., television broadcasters) [31]. Current research suggests, rather, that media personae may no longer be restricted to macro celebrities but may include ordinary people owing to the expanded accessibility of short-form video platforms. Viewers’ well-being can, therefore, more easily be invoked because they are more likely to encounter peer networks where they can be understood and socially supported [21]. Additionally, recovery (e.g., from feelings of hopelessness) effects can be enhanced because of increased interactions on short-form video platforms. The concept of a PSR was proposed by Horton and Wohl [1] at a time when it was impossible for viewers to communicate with media personae in real-time on screen. Now, however, short-form video platforms afford nearly synchronous communication. For example, viewers can participate in a live conversation and receive almost instant responses from media personae through live streaming. This can empower viewers because the replies of media personae are tailored to their situational requirements. Consequently, PSRs developed on short-form video platforms can be a crucial means of recovery for many users.

Third, our findings indicate that perceived similarities had a significant moderation effect on the relationship between hopelessness and PSRs and the indirect effect of perceived hopelessness on self-identity/well-being. This suggests that the similarity-attraction mechanism also exists in short-form video platforms. When hopeless individuals with high perceived similarities use short-form videos, the more they feel hopelessness, the more inclined they are to establish PSRs. In summary, the extent of perceived similarities moderates the correlation between hopelessness and PSRs on short-form video platforms, a finding that extends research on perceived similarity.

### Practical implications and suggestions

This study offers new insights into the PSR mechanisms for short-form video platforms. These insights have practical implications for both platform designers and multi-channel networks. Specifically, we explored the potentially positive role that PSRs can play on short-form video platforms in assisting individuals experiencing hopelessness. To optimize this role for users, short-form video platforms can adapt their algorithms to increase recommendations for more similar media personae. As noted above, PSRs on short-form video platforms often form when users perceive high levels of similarity with a media persona or another user. It is therefore important for short-form video platforms to be aware of this unique characteristic and improve their recommendation algorithms.

Furthermore, our research can inform communication strategies for live streamers. Users experiencing hopelessness reportedly engage in PSRs on short-form video platforms to learn from media personae and improve their psychological health. In view of this, a multi-channel network could reach a wider target audience by including those who suffer from hopelessness, thereby expanding their range of followers of the platform. To attract and retain this group of fans, multi-channel networks can create training manuals to instruct live streamers about acknowledging their viewers’ precise needs and thereby build a more immersive interactive environment with users who feel hopeless. When live streamers start synchronous communication via live streaming, communication should focus on listening rather than talking. A feasible way to achieve this is to record all synchronous comments and address issues of great concern among viewers as the next live-streaming topic. Only when viewers notice that a live streamer identifies with their personal issues and provides personalized suggestions will a PSR begin to form. Overall, live streamers should be encouraged to care more about their viewers and provide a more interactive environment.

While this study demonstrates that PSRs may serve as a supportive mechanism through which individuals experiencing hopelessness can strengthen their sense of self-identity and well-being, it is also important to acknowledge that PSRs are not inherently or universally beneficial. Depending on the characteristics of the PSR—such as its intensity, duration, and the viewer’s psychological vulnerability—such relationships may also contribute to adverse outcomes, including siloing [75], escapism [76], or identity distortion [77], isolation [78] and ideological rupturing [79]. Future research should critically examine the boundary conditions under which PSRs transition from offering psychological support to reinforcing maladaptive emotional or cognitive patterns.

### Limitations and future studies

This study had some limitations that can be improved through further research. First, the present study did not use video clips as stimuli. This is because PSRs are unique to the individual, defined under personal conditions, and can be affected by media offerings and recipients’ motivations [31]. Thus, it would be difficult to use a single video clip as a universal stimulus. However, it would be valuable to employ video clips in future studies, especially when the focus of the study examines the extent to which specific functions exert effects on PSRs.

Second, the present study used a survey to presume and evaluate the mediation model, which may have implied causality. However, the causality was not established through a well-controlled experimental setting. Future studies should acknowledge this limitation and employ a design that is more conducive to determining causality.

Third, the study is based on cross-sectional survey data, which limits the ability to make causal inferences regarding the psychological mechanisms proposed. Future research should incorporate longitudinal or experimental designs to better capture the temporal dynamics of PSR development and its effects. Such approaches would also allow for a more nuanced understanding of when and how PSRs exert beneficial—or potentially harmful—impacts on users’ psychological states.

Fourth, the analytical significance of this study might be due to the large sample size rather than the true effects in the study limitation section. Future research can build upon these findings for further investigation and validation.

## Conclusion

This study offers an initial perspective on how positive health effects may be independently attained by users of short-form video platforms through PSRs. Based on the theoretical model of the development of PSRs, we examined the mediating role of PSRs for users experiencing a sense of hopelessness to improve their self-identity and well-being [34]. Additionally, we investigated the role of perceived similarity in moderating the relationship between hopelessness and PSR and the indirect of perceived similarities on self-identity/well-being. This study’s findings offer insights into both the hopelessness theory of depression and PSR theory within the context of short-form video use. Furthermore, the study provides practical implications for short-form video platforms and live-streamers.

## Data Availability

No datasets were generated or analysed during the current study.

## References

[1] Horton D, Richard WR. Mass communication and para-social interaction: observations on intimacy at a distance. Psychiatry. 1956;19(3):215–29. 13359569 10.1080/00332747.1956.11023049

[2] Hartmann T, Goldhoorn C. Horton and Wohl revisited: exploring viewers’ experience of parasocial interaction. J Communication. 2011;61(6):1104–21.

[3] Dibble JL, Hartmann T, Rosaen SF. Parasocial interaction and parasocial relationship: conceptual clarification and a critical assessment of measures. Hum Commun Res. 2016;42(1):21–44.

[4] Li W, Ding H, Xu G, Yang J. The impact of fitness influencers on a social media platform on exercise intention during the COVID-19 pandemic: the role of parasocial relationships. Int J Environ Res Public Health. 2023;20(2):1113. 36673868 10.3390/ijerph20021113 PMC9858650

[5] Johnson EK, Rothermich K, Shoenberger H. I’ll have what she’s having: parasocial communication via social media influences on risk behavior. J Social Media Soc. 2020;9(2):319–34.

[6] Siegert RJ, Narayanan A, Dipnall J, Gossage L, Wrapson W, Sumich A, Tautolo ES. Depression, anxiety and worry in young Pacific adults in new Zealand during the COVID-19 pandemic. Australian New Z J Psychiatry. 2023;57(5):698–709. 35957548 10.1177/00048674221115641

[7] Abramson LY, Alloy LB, Hogan ME, Whitehouse WG, Gibb BE, Hankin BL, Cornette MM. The hopelessness theory of suicidality. In: Joiner T, Rudd MD, editors. Suicide science: expanding the boundaries. Norwell: Kluwer Academic; 2002. pp. 17–32.

[8] Zuo B, Yang K, Yao Y, Han S, Nie S, Wen F. The relationship of perceived social support to feelings of hopelessness under COVID-19 pandemic: the effects of epidemic risk and meaning in life. Pers Indiv Differ. 2021;183:111110. 10.1016/j.paid.2021.111110 PMC8416552 34511679

[9] Sakib MN, Zolfagharian M, Yazdanparast A. Does parasocial interaction with weight loss vloggers affect compliance? The role of vlogger characteristics, consumer readiness, and health consciousness. J Retailing Consumer Serv. 2020;52:e101733.

[10] Sokolova K, Perez C. You follow fitness influencers on YouTube. But do you actually exercise? How parasocial relationships, and watching fitness influencers, relate to intentions to exercise. J Retailing Consumer Serv. 2021;58:e102276.

[11] Rubin RB, McHugh MP. Development of parasocial interaction relationships. J Broadcast Electron Media. 1987;31(3):279–92.

[12] Tukachinsky R, Walter N, Saucier CJ. Antecedents and effects of parasocial relationships: A meta-analysis. J Communication. 2020;70(6):868–94.

[13] Berger CR, Calabrese RJ. Some explorations in initial interaction and beyond: toward a developmental theory of interpersonal communication. Hum Commun Res. 1974;1(2):99–112.

[14] Baumeister RF. The self. In: Baumeister RF, Finkel EJ, editors. Advanced social psychology: the state of the science. New Yorok: McGraw-Hill; 2010. pp. 139–75.

[15] CNNIC. The 50th China Statistical Report on Internet Development. (online). 2022. http://www.cnnic.net.cn/NMediaFile/2022/1020/MAIN 16662-586615125EJOL1VKDF.pdf. Accessed 10th September 2022.

[16] Lin J, de Kloet J. Platformization of the unlikely creative class: Kuaishou and Chinese digital cultural production. Social Media + Soc. 2019;5(4):1–12.

[17] Zulli D, Zulli DJ. Extending the internet meme: conceptualizing technological mimesis and imitation publics on the TikTok platform. New Media Soc. 2020;24(8):1–19.

[18] Vizcaíno-Verdú A, Abidin C. Music challenge memes on tiktok: Understanding in-group storytelling videos. Int J Communication. 2022;16:883–908.

[19] Nah HS. The appeal of real in parasocial interaction: the effect of self-disclosure on message acceptance via perceived authenticity and liking. Comput Hum Behav. 2022;134:e107330.

[20] Wang Y. Humor and camera view on mobile short-form video apps influence user experience and technology-adoption intent, an example of TikTok (DouYin). Comput Hum Behav. 2020;110:e106373.

[21] Eriksson Krutrök M. Algorithmic closeness in mourning: vernaculars of the hashtag# grief on TikTok. Social Media + Soc. 2021;7(3):1–12.

[22] Abramson LY, Metalsky GI, Alloy LB. Hopelessness depression: A theory-based subtype of depression. Psychol Rev. 1989;96(2):358–72.

[23] Aalbers G, McNally RJ, Heeren A, De Wit S, Fried EI. Social media and depression symptoms: A network perspective. J Exp Psychol Gen. 2019;148(8):1454. 30507215 10.1037/xge0000528

[24] Kubey RW. Television and aging: Past, present, and future. Gerontologist. 1980;20(1):16–35. 6991371 10.1093/geront/20.1.16

[25] Katz E, Blumler JG, Gurevitch M. Uses and gratifications research. Public Opin Q. 1973;37(4):509–23.

[26] Rubin AM. Uses and gratifications. The SAGE handbook of media processes and effects. Thousand Oaks, CA: Sage; 2009. pp. 147–59.

[27] Rubin AM, Perse EM, Powell RA. Loneliness, parasocial interaction, and local television news viewing. Hum Commun Res. 1985;12(2):155–80.

[28] Goldsmith DJ. The role of Facebook in supportive communication. In: Burleson BR, Albrecht TL, Sarason IG, editors. Communication of social support: Messages, interactions, relationship and community. Thousand Oaks, CA: Sage; 1994. pp. 29–49.

[29] Newton BJ, Buck EB. Television as significant other: its relationship to self-descriptors in five countries. J Cross-Cult Psychol. 1985;16(3):289–312.

[30] Horton D, Strauss A. Interaction in audience-participation shows. Am J Sociol. 1957;62(6):579–87.

[31] Schramm H, Wirth W. Testing a universal tool for measuring parasocial interactions across different situations and media. J Media Psychol. 2010;22(1):26–36.

[32] Hoffner C, Cohen EL. Mental health-related outcomes of robin williams’ death: the role of parasocial relations and media exposure in stigma, help-seeking, and outreach. Health Commun. 2018;33(12):1573–82. 29048251 10.1080/10410236.2017.1384348

[33] Song I, Larose R, Eastin MS, Lin CA. Internet gratifications and internet addiction: on the uses and abuses of new media. Cyberpsychology Behav. 2004;7(4):384–94. 10.1089/cpb.2004.7.384 15331025

[34] Tukachinsky R, Stever G. Theorizing development of parasocial engagement. Communication Theory. 2019;29(3):297–318.

[35] Knapp ML. Social intercourse: from greeting to goodbye. Boston, MA: Allyn & Bacon, Incorporated;; 1978.

[36] Knapp ML, Vangelisti AL, Caughlin JP. Interpersonal communication and human relationships. New York: Pearson Higher Ed; 2014.

[37] Cohen J. Defining identification: A theoretical look at the identification of audiences with media characters. Mass Communication Soc. 2001;4(3):245–64.

[38] Katz E, Foulkes D. On the use of the mass media as escape: clarification of a concept. Pub Opin Q. 1962;26(3):377–88.

[39] Harwood J. Age identification, social identity gratifications, and television viewing. J Broadcast Electron Media. 1999;43(1):123–36.

[40] Leith AP. Parasocial cues: the ubiquity of parasocial relationships on twitch. Communication Monogr. 2021;88(1):111–29.

[41] Markus H, Kunda Z. Stability and malleability of the self-concept. J Personal Soc Psychol. 1986;51(4):858. 10.1037//0022-3514.51.4.858 3783430

[42] Cohen J, Appel M, Slater MD. Media, identity, and the self. In: Oliver M, B, Raney AA, Bryant J, editors. Media effects. London: Routledge; 2019. pp. 179–94.

[43] Ochse R, Plug C. Cross-cultural investigation of the validity of erikson’s theory of personality development. J Personal Soc Psychol. 1986;50(6):1240–52.

[44] Mann M, Hosman CMH, Schaalma HP, de Vries NK. Self-esteem in a broad-spectrum approach for mental health promotion. Health Educ Res. 2004;19(4):357–72. 15199011 10.1093/her/cyg041

[45] Ellemers N, Spears R, Doosje B. Self and social identity. Ann Rev Psychol. 2002;53(1):161–86. 11752483 10.1146/annurev.psych.53.100901.135228

[46] Gabriel S, Young AF, Naidu E, Schneider V. How parasocial relationships affect our self-concepts. In: Tukachinsky R, editor. The Oxford handbook of parasocial experiences. Oxford University Press; 2023. pp. 252–68. (1ª.

[47] Moyer-Gusé E. Toward a theory of entertainment persuasion: explaining the persuasive effects of entertainment-education messages. Communication Theory. 2008;18(3):407–25.

[48] Tsay M, Bodine BM. Exploring parasocial interaction in college students as a multidimensional construct: do personality, interpersonal need, and television motive predict their relationships with media characters? Psychol Popular Media Cult. 2012;1(3):185.

[49] Bhandari A, Bimo S. Why’s everyone on TikTok now? The algorithmized self and the future of self-making on social media. Social media + Soc. 2022;8(1):e20563051221086241.

[50] Hartmann T. Parasocial interaction, parasocial relationships, and well-being. In: Reinecke L, Oliver MB, editors. The Routledge handbook of media use and well-being: international perspectives on theory and research on positive media effects. London: Routledge/Taylor & Francis Group; 2017. pp. 131–44.

[51] Diener E, Suh EM, Lucas RE, Smith HL. Subjective well-being: three decades of progres*s*. Psychol Bull. 1999;125(2):276–302.

[52] Ryan RM, Deci EL. Self-determination theory and the facilitation of intrinsic motivation, social development, and well-being. Am Psychol. 2000;55:68–78. 11392867 10.1037//0003-066x.55.1.68

[53] Keyes CL. The mental health continuum: From languishing to flourishing in life. Journal of health and social behavior. 2002; 207 – 22. 12096700

[54] Seligman ME. Positive psychology, positive prevention, and positive therapy. Handb Posit Psychol. 2002; 3–12.

[55] Sood S, Rogers EM. Dimensions of parasocial interaction by letter-writers to a popular entertainment-education soap Opera in India. J Broadcast Electron Media. 2000;44(3):386–414.

[56] Nathan W, Emily AA, Tukachinsky R. Initiation and evolution of PSRs. In: Tukachinsky R, editor. The Oxford handbook of parasocial experiences. Oxford University Press; 2023. pp. 125–46. (1ª.

[57] Wang Q, Fink EL, Cai DA. Loneliness, gender, and parasocial interaction: A uses and gratifications approach. Communication Q. 2008;56(1):87–109.

[58] Bond BJ. Parasocial relationships with media personae: why they matter and how they differ among heterosexual, lesbian, gay, and bisexual adolescents. Media Psychol. 2018;21(3):457–85.

[59] Ross L, Greene D, House P. The false consensus effect: an egocentric bias in social perception and attribution processes. J Exp Soc Psychol. 1977;13(3):279–301.

[60] Baldwin MW. Relational schemas and the processing of social information. Psychol Bull. 1992;112(3):461–84.

[61] Klimmt C, Hartmann T, Schramm H. Parasocial interactions and relationships. In: Byrant J, Vorderer P, editors. Psychology of entertainment. NY: Routledge; 2006. pp. 291–313.

[62] Paravati E, Naidu E, Gabriel S, Wiedemann C. More than just a tweet: the unconscious impact of forming parasocial relationships through social media. Psychol Consciousness: Theory Res Pract. 2020;7(4):388–403.

[63] CNNIC. The 47th China Statistical Report on Internet Development. (online). 2022. https://www.cac.gov.cn/2021-02/03/c_1613923423079314.htm. Accessed 10 February 2021.

[64] Li D, Li X, Wang Y, Bao Z. Parenting and Chinese adolescent suicidal ideation and suicide attempts: the mediating role of hopelessness. J Child Fam Stud. 2016;25(5):1397–407.

[65] Bolland JM, McCallum DM, Lian B, Bailey CJ, Rowan P. Hopelessness and violence among inner-city youths. Matern Child Health J. 2001;5(4):237–44. 11822525 10.1023/a:1013028805470

[66] Xing ZJ. Short-version of the Chinese urban residents’ well-being scale. Chin J Behav Med Brain Sci. 2003; 06. The full reference of this paper in Chinese is 邢占军.(2003).中国城市居民主观幸福感量表简本的编制.中国行为医学科学(06).

[67] Auter PJ, Palmgereen P. Development and validation of a parasocila interaction measure: the audience-persona interaction scale. Communication Res Rep. 2000;17(1):79–89.

[68] Bai QY, Bai S, HuangY, Hsueh FH, Wang P. Family incivility and cyberbullying in adolescence: A moderated mediation model. Comput Hum Behav. 2020; 110, e106315.

[69] Chen X, Li S. Serial mediation of the relationship between impulsivity and suicidal ideation by depression and hopelessness in depressed patients. BMC Public Health. 2023;23(1):1457. 37525167 10.1186/s12889-023-16378-0 PMC10388524

[70] Baek YM, Bae Y, Jang H. Social and parasocial relationships on social network sites and their differential relationships with users’ psychological well-being. Cyberpsychology Behav Social Netw. 2013;16(7):512–7. 10.1089/cyber.2012.0510 23697533

[71] Liu PL. Parasocial relationship in the context of the COVID-19 pandemic: A moderated mediation model of digital media exposure on political trust among Chinese young people. Comput Hum Behav. 2023;141:107639. 10.1016/j.chb.2022.107639 PMC9794563 36589719

[72] Hayes AF. PROCESS: A versatile computational tool for observed variable mediation, moderation, and conditional process modeling (Online). 2012. http://www.afhayes.com/public/process2012.pdf. Accessed 10 September 2022.

[73] Hayes AF, Scharkow M. The relative trustworthiness of Inferential tests of the indirect effect in statistical mediation analysis: does method really matter? Psychol Sci. 2013;24(10):1918–27. 23955356 10.1177/0956797613480187

[74] Panzarella C, Alloy LB, Whitehouse WG. Expanded hopelessness theory of depression: on the mechanisms by which social support protects against depression. Cogn Therapy Res. 2006;30(3):307–33.

[75] Radu RN. How I learned to hate you. Parasocial interactions in echo chambers and their spillover effects. Methaodos Revista De Ciencias Sociales. 2023;11(1):1–16.

[76] de Bérail P, Bungener C. Parasocial relationships and YouTube addiction: the role of viewer and YouTuber video characteristics. Psychol Lang Communication. 2022;26(1):169–206.

[77] Wen N. Celebrity influence and young people’s attitudes toward cosmetic surgery in singapore: the role of parasocial relationships and identification. Int J Communication. 2017;11(19):1234–52.

[78] Liu J, Lee JS. Social media influencers and followers’ loneliness: the mediating roles of parasocial relationship, sense of belonging, and social support. Online Media Global Communication. 2024;3(4):607–30.

[79] Jones S, Cronin J, Piacentini MG. Celebrity brand break-up: fan experiences of para-loveshock. J Bus Res. 2022;145:720–31.

